# Wavelet-Based Health Monitoring Approach for Train Door Actuation Using Motor Current Analysis

**DOI:** 10.3390/s26092898

**Published:** 2026-05-06

**Authors:** Yaojung Shiao, Premkumar Gadde, Manichandra Bollepelly

**Affiliations:** 1 Department of Vehicle Engineering, National Taipei University of Technology, Taipei 10608, Taiwan; 2 Railway Vehicle Research Center, National Taipei University of Technology, Taipei 10608, Taiwan; 3 Comtrend Corporation, New Taipei City 241405, Taiwan

**Keywords:** health monitoring, train door system, obstacle detection, motor current signal, discrete wavelet transform

## Abstract

Train door actuation systems are critical safety components in railway vehicles, where early fault detection is essential for safe operation and reduced service disruptions. Conventional monitoring approaches often rely on additional sensors such as infrared detectors or vision systems, which increase system complexity and cost. To overcome these limitations, this study proposes a wavelet-based health monitoring structure for detecting electrical and mechanical faults using motor current signal analysis. A dynamic model of the train door actuation mechanism, including a DC motor, gearbox, and lead screw, was developed in MATLAB/Simulink to simulate conditions such as armature electrical faults, brush wear, increased friction, and lead screw misalignment. Motor current signals were analyzed using the Discrete Wavelet Transform with a Daubechies (db10) mother wavelet to extract diagnostic features based on the L1-norms of wavelet coefficients at levels W8 and W9 along with the motor starting current peak. Experimental validation using a LabVIEW-based test platform demonstrated fault detection accuracy above 96% with a response time below 0.3 s, confirming the effectiveness of the proposed approach for predictive maintenance of railway door systems.

## 1. Introduction

Train door systems are among the most critical safety subsystems in modern railway vehicles, as they directly influence passenger safety, reliable boarding operations, and accident prevention during train movement. Reports from railway operations indicate that nearly 30–40% of service delays and operational failures are related to malfunctions in door actuation and control mechanisms [1]. Such failures commonly arise from mechanical degradation such as component wear and increased friction in transmission elements, as well as electrical faults within the DC motor drive system. These issues can reduce operational efficiency and more importantly introduce potential safety risks while affecting the overall reliability of the railway system.

To reduce accidents associated with door operation, various sensor-based obstacle detection systems have been implemented in railway door mechanisms. Traditional approaches typically employ infrared sensors or sensitive edge sensors to detect physical obstructions during the door closing. Although effective, these systems require additional hardware, periodic calibration, and are often influenced by environmental conditions. More recently, vision-based monitoring methods using deep learning have been explored. For example, Tan et al. proposed a Deep Differentiation Segmentation Neural Network (DDSNN) capable of detecting foreign objects under complex environments, while Liu et al. introduced the AnoDet framework achieving detection accuracy above 97% [2,3]. Similarly, Yan et al. developed a deep learning model for object detection in metro systems [4]. Despite their promising accuracy, these approaches depend on camera systems and intensive computation, making them sensitive to lighting conditions, occlusion, and high processing requirements.

As an alternative, current-based monitoring methods have gained attention due to their simplicity and cost-effectiveness. These methods utilize the relationship between mechanical load variations and motor current response [5]. Early work by Liu et al. demonstrated back-EMF-based fault detection, while studies by Michal et al. and Li et al. showed that ripple components in motor current signals can indicate obstruction events [6,7,8]. Although effective, these approaches are often sensitive to noise and primarily rely on time-domain or frequency-domain analysis, which may not fully capture transient and non-stationary characteristics of motor current signals.

Motor current analysis has been widely applied in various applications, including anti-pinch protection in automotive power windows and electrical machine diagnostics, where it has demonstrated effectiveness in detecting both electrical and mechanical anomalies [9,10,11]. However, many of these approaches rely primarily on time-domain or frequency-domain analysis, which may not adequately capture the transient and non-stationary characteristics of motor current signals, particularly in dynamic systems such as train door mechanisms.

To address these limitations, wavelet-transform-based methods have been increasingly adopted for analyzing non-stationary signals in electromechanical systems. The multiresolution analysis framework introduced by Mallat enables the decomposition of signals into multiple frequency bands, allowing localized feature extraction in both time and frequency domains [12]. Building on this concept, the Discrete Wavelet Transform (DWT) has been applied for motor fault diagnosis. For example, Liu et al. improved ripple signal clarity using wavelet thresholding techniques, while Wu et al. demonstrated the effectiveness of wavelet-based diagnostics in a train door test platform [13,14].

In addition, Motor Current Signature Analysis (MCSA) has been recognized as a non-intrusive and practical diagnostic method. Studies by Miljkovic and Majdi have shown its capability in detecting electrical faults without requiring additional sensors. Nevertheless, these approaches are generally focused on steady-state conditions and have limited effectiveness in capturing transient fault behavior [15,16].

Further advancements in signal processing techniques have enhanced diagnostic capabilities. The development of orthogonal wavelet bases by Daubechies enabled improved time–frequency localization, facilitating the detection of short-duration disturbances associated with electrical faults and load variations [17]. Subsequent work by Douglas et al. introduced wavelet-based transient analysis methods that demonstrated higher sensitivity compared to traditional spectral techniques [18]. Despite these advancements, most studies have been limited to industrial motor applications and do not fully address complex electromechanical systems such as railway door mechanisms, where multiple components interact simultaneously.

Recent research in condition monitoring has increasingly explored data-driven and deep learning-based approaches, which have shown strong performance in complex detection tasks. However, these methods typically require large datasets and high computational resources, and often operate as black box models, limiting their interpretability and practical implementation in real-time railway systems. In contrast, signal-based and physics-oriented approaches provide greater transparency and lower computational complexity, making them more suitable for embedded applications.

Another important consideration in modern monitoring systems is the handling of uncertainty. Advanced techniques, such as Bayesian inference-based frameworks, explicitly model uncertainties in system behavior and measurements to improve diagnostic reliability [19]. While effective, these methods often involve increased modeling complexity and require accurate prior knowledge of system parameters.

Moreover, explainability has become a critical requirement in safety critical applications such as railway systems. Many advanced data-driven approaches lack clear physical interpretation, making it difficult to relate diagnostic outcomes to actual system behavior and limiting their usefulness for maintenance decision-making.

In this context, the proposed approach adopts a signal-based and physically interpretable framework for fault diagnosis. Instead of relying on data-intensive models, the method utilizes motor current signals combined with wavelet-based feature extraction to identify fault conditions. The selected features, including W8 and W9 coefficients and motor starting current characteristics, provide clear physical meaning and enable direct interpretation of electromechanical fault mechanisms. Furthermore, the proposed framework is computationally efficient and sensorless, making it suitable for real-time implementation in practical railway systems.

Additional studies have explored enhanced wavelet thresholding techniques to improve noise reduction and feature extraction from motor current signals [20]. However, these investigations primarily focus on industrial electric machines, while limited attention has been given to complex electromechanical systems such as railway door mechanisms, which involve interacting components including gearboxes, lead screws, rollers, and sliding door assemblies [21,22,23]. Furthermore, research by Teel-Jongebloed demonstrated that degradation mechanisms such as brush wear in DC motors can be identified through statistical analysis of current signals, highlighting the potential of current-based monitoring for detecting combined electrical and mechanical faults [13].

To provide a clearer understanding of existing approaches, Table 1 summarizes key related studies along with their main contributions and associated limitations. It can be observed that vision-based methods achieve high detection accuracy. However, they rely on additional hardware and are often sensitive to environmental conditions such as lighting variations and occlusion. In contrast, current-based approaches offer a simpler and more cost-effective alternative, but many existing methods are primarily focused on steady-state or post-fault detection and exhibit limited capability in capturing transient and early-stage degradation. Furthermore, most studies address electrical and mechanical faults independently and are not specifically designed for integrated electromechanical systems such as train door mechanisms.

From this analysis, several research gaps become evident. There is a lack of sensor-less approaches capable of simultaneously detecting both electrical and mechanical faults, along with limited emphasis on early-stage or pre-diagnostic fault detection. In addition, existing methods do not sufficiently exploit time–frequency techniques to capture transient motor current behavior, and there remains limited application of such approaches to complex railway door actuation systems.

To address the identified research gaps, this study proposes a wavelet-based health monitoring framework for train door actuation systems using motor current analysis. The proposed approach enables the simultaneous detection of both electrical and mechanical faults without the need for additional sensors. By focusing on the W8 and W9 wavelet coefficients along with motor starting current characteristics, the method provides physically meaningful indicators that allow early identification of system degradation. The framework is designed to support pre-diagnostic monitoring, enabling faults to be detected before they lead to failure. Its effectiveness is validated through experimental implementation using a real-time LabVIEW-based test platform. In addition, the integration of the proposed method with Failure Mode and Effects Analysis supports maintenance prioritization. Overall, the approach offers a practical, cost-effective, and interpretable solution for improving reliability and predictive maintenance in railway door systems.

## 2. Methodology and Simulation

The proposed framework integrates simulation modeling, wavelet signal processing, feature extraction, and rule-based diagnostic classification. The process includes: motor current acquisition, wavelet decomposition, extraction of W8 and W9 coefficients and MSC peak, threshold comparison, and fault classification.

### 2.1. Overview of Fault Simulation in Train Door System

The proposed health monitoring (pre-diagnostic) framework was developed to identify early-stage electrical and mechanical faults in train door actuation systems before they progress to major failures. The methodology integrates a MATLAB/Simulink-based simulation environment with Discrete Wavelet Transform-based feature extraction to analyze motor current signals as indicators of system health.

By analyzing variations in motor current waveforms, the proposed framework effectively distinguishes between normal operating conditions, electrical anomalies, and mechanical degradation within the train door actuation system. The electrical subsystem, which includes the DC motor and its control circuitry, reflects faults such as armature short or open circuits through abnormal current signatures. In contrast, the mechanical subsystem, comprising the gearbox, lead screw, roller assembly, and sliding door panels, exhibits characteristic current fluctuations when frictional resistance increases due to wear or misalignment. These distinct waveform patterns enable accurate differentiation of fault sources and severities within the integrated diagnostic model.

Electrical faults were simulated as armature short and open circuits, while mechanical faults were modeled by gradual increases in frictional torque beyond the nominal operating limit. Each fault scenario generated unique current signatures, which were decomposed using DWT to extract discriminative time–frequency features for health monitoring analysis.

#### 2.1.1. Electrical Fault Simulation in Train Door Actuation System

Electrical faults in the train door actuation system were simulated by varying the armature circuit parameters of the DC motor to emulate realistic degradation and failure conditions, including coil shorting, open winding, and progressive deterioration. The motor starting current was adopted as the principal health indicator, as it reflects the electrical integrity of the armature during each door closing cycle. Under normal operation, the MSC remains within a specified tolerance band and any significant deviation indicates potential electrical malfunction.

##### Armature Health Monitoring

The armature health status was evaluated based on the MSC profile. Monitoring MSC enables early maintenance intervention before critical failure occurs. The MSC is influenced by several factors, including variations in armature resistance, short circuits, open circuits, loose couplings, or eccentricity-related faults. In the developed simulation model of the train door control system, MSC was recorded under healthy operating conditions to establish baseline limits. During each door closing event, the pre-diagnostic algorithm checks whether the measured MSC falls within this healthy threshold. If the MSC deviates from the nominal range, the system sequentially evaluates for open-circuit and short-circuit faults. Cases that do not match either condition are classified as unidentified electrical faults.

##### Armature Short-Circuit Fault

**Figure 1:**
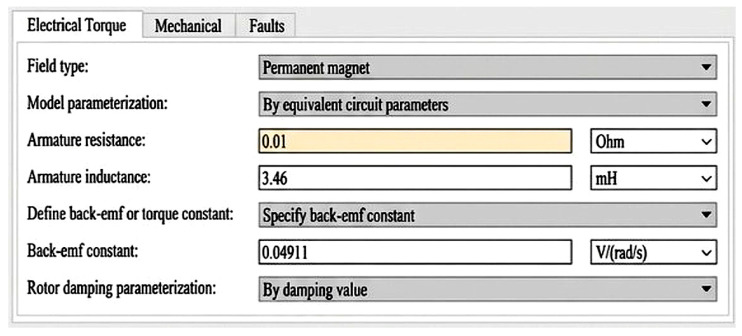
Parameters used to simulate the armature short-circuit fault in the DC motor.

A short-circuit fault in the DC motor represents insulation breakdown or partial winding failure, which drastically reduces the armature resistance. In the simulation, this condition was modeled by setting the armature resistance to 0.01 Ω, resulting in an abnormal surge in current as shown in Figure 1.

##### Armature Open-Circuit Fault

**Figure 2:**
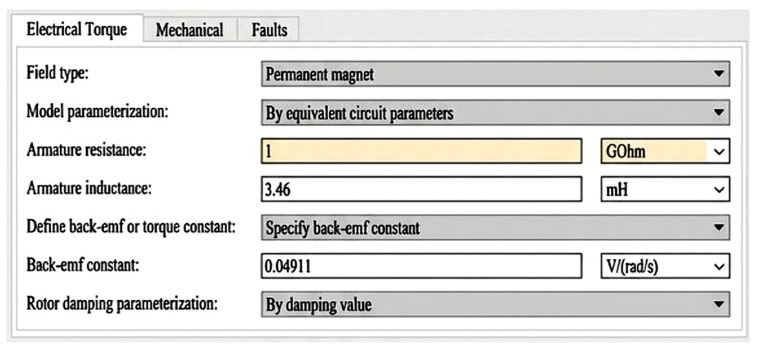
Parameters used to simulate the armature open-circuit fault in the DC motor.

An open-circuit fault was simulated to represent coil breakage or disconnected windings. This fault leads to a significant increase in armature resistance, modeled as 1 GΩ in the simulation as shown in Figure 2.

**Electromechanical Dynamics**

The motor dynamic behavior followed standard electromechanical relations expressed as

(1) $$T=J\frac{d\omega }{dt}+b\omega +T_{L}$$

(2) $$V=L\frac{di}{dt}+Ri+e_{b}$$

where *T* is the electromagnetic torque, *i* is the armature current, *J* is the rotor inertia, *b* is the viscous damping coefficient, *T_L_* is the load torque, and *e_b_* is the back electromotive force.

**Progressive Degradation Modeling**

To emulate gradual degradation modes, additional fault scenarios were incorporated into the simulation:

- Brush wear: modeled as a time varying armature resistance *R*(*t*).
- Bearing friction increase: represented by an increased damping coefficient b.
- Commutator imbalance: periodic fluctuation in the torque constant *K_t_.*

Each fault condition produced distinct distortions in the motor current waveform, such as amplitude ripple variations and transient spikes. These temporal features were analyzed using the Discrete Wavelet Transform to extract sensitive time–frequency characteristics corresponding to early-stage electrical degradation. The DWT coefficients and MSC trends served as diagnostic parameters within the proposed health monitoring (pre-diagnostic) algorithm, which accurately classified the armature condition and generated alerts for short circuit, open circuit, or unidentified anomalies when predefined thresholds were exceeded.

#### 2.1.2. Mechanical Fault Simulation in Train Door Actuation System

The mechanical subsystem of the train door actuation model consisted of the gearbox, lead screw mechanism, roller assembly, and sliding door panels. Mechanical degradation was simulated by varying the Coulomb friction parameter in the actuation model to emulate the effects of wear, contamination, and misalignment. Under healthy operating conditions, the nominal friction torque was maintained at 0.173 Nm. A gradual increase to 0.20 Nm represented moderate degradation caused by roller wear, dust accumulation, or minor lead screw misalignment. The severe fault condition, defined at 0.29 Nm, simulated excessive friction beyond the system’s allowable limit, resulting in door jamming or incomplete closure.

The health monitoring (pre-diagnostic) algorithm was configured to tolerate frictional growth up to 0.27–0.28 Nm. Beyond this threshold, a “friction higher than threshold” alert was triggered to initiate maintenance intervention before operational failure occurred. The lead screw efficiency (η) was calculated as

(3) $$\eta =\frac{F\times P}{2\pi T}$$

where *F* is the thrust force, *P* is the lead screw pitch, and *T* is the applied torque.

A reduction in efficiency indicated increasing frictional losses or misalignment within the mechanical transmission path. To replicate realistic degradation scenarios, additional mechanical faults were introduced:

- Panel misalignment: modeled as an added side-load torque on the lead screw;
- Gear wear: represented by a reduction in transmission efficiency.

##### Friction Fault Detection

**Figure 3:**
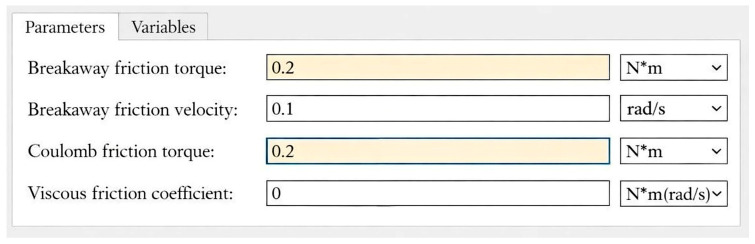
Simulation parameters of frictional increase in the door actuation system.

An increase in door friction directly affects the motor current response, making it an effective diagnostic signature. In the simulation model, the Coulomb friction parameter was gradually increased from 0.173 Nm to 0.20 Nm to emulate the early stage of frictional fault as shown in Figure 3. The pre-diagnostic algorithm was designed to tolerate this increase up to 0.27 Nm, beyond which performance degradation became critical.

##### Friction Higher than Threshold Level

**Figure 4:**
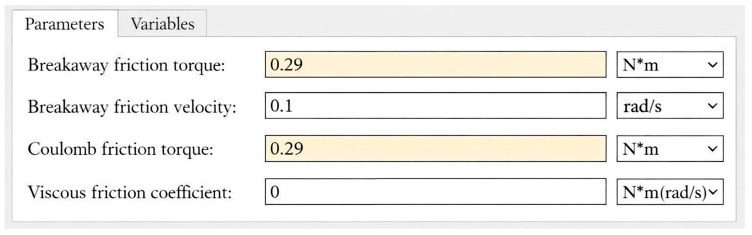
Simulation parameters of friction exceeding the threshold level.

When friction exceeded the allowable limit, the door operation was automatically restricted to prevent further damage. This severe fault was modeled by increasing both breakaway and Coulomb friction parameters to 0.29 Nm, corresponding to excessive resistance in the drive mechanism as shown in Figure 4.

Each mechanical fault condition generated distinct current waveform distortions, serving as diagnostic indicators of mechanical deterioration. These current signals were analyzed using the Discrete Wavelet Transform to extract time–frequency features sensitive to progressive mechanical faults. The resulting wavelet-based features enabled the health monitoring (pre-diagnostic) algorithm to identify frictional growth trends, gear wear, and alignment-related anomalies at an early stage, thereby ensuring predictive maintenance and reliable operation of the train door system.

### 2.2. Feature Extraction Using Wavelet Transform

Motor current signals were analyzed using the Discrete Wavelet Transform (DWT) to extract transient and steady-state features associated with electrical and mechanical faults. The Daubechies (db10) mother wavelet was selected due to its effective time–frequency localization capability for non-stationary current signals. The current waveform was decomposed into nine levels (W1–W9), and the L_1_-norm of the detailed coefficients at each level was computed to quantify the signal energy distribution.

Lower decomposition levels (W1–W4) capture high-frequency components, mainly due to switching noise, electrical transients, and measurement disturbances. Intermediate levels (W5–W7) reflect a mix of electrical and mechanical effects with moderate diagnostic relevance. Higher levels (W8 and W9) represent low-frequency components related to electromechanical behavior, such as load torque variations, friction growth, and armature degradation. Since early-stage mechanical faults and gradual electrical deterioration primarily manifest in these low-frequency currents, W8 and W9 serve as physically meaningful indicators of system health rather than mere mathematical constructs.

The resulting L_1_-norm values under friction increase, armature short-circuit, and armature open-circuit conditions are summarized in Table 2, enabling a comparative assessment of fault sensitivity across decomposition levels.

Among all decomposition levels, the L_1_-norm values corresponding to the eighth and ninth decomposition levels exhibited the most pronounced and consistent variations under both mechanical and electrical fault conditions. In particular, an increase in friction torque resulted in a significant rise in the energy content of the level-9 coefficients, reflecting dominant low-frequency load modulation effects, whereas armature short- and open-circuit faults produced distinct changes in the energy content of both level-8 and level-9 coefficients, accompanied by abnormal motor starting current peak amplitudes. Accordingly, the L_1_-norm of W8 and W9 was employed to detect frictional and electromechanical disturbances, while the MSC peak was used as a complementary indicator of armature integrity. The combined use of MSC peak, W8, and W9 thus provides a compact and physically interpretable multi-domain feature set for reliable fault identification in the proposed health monitoring (pre-diagnostic) framework.

The energy norm for each level was computed as

(4) $$W_{n}=\sqrt{\sum_{i}|}D_{n,i}{|}^{2}$$

where *D_n,i_* denotes the detailed coefficients at level *n*. Elevated *W_n_* values or abnormal MSC peaks indicated possible electrical or mechanical degradation.

Distinct feature patterns were observed across different fault types:

- Armature short circuit: sharp rise in MSC peak with high W8 and W9 norms.
- Armature open circuit: reduced amplitudes across all features due to current discontinuity.
- Frictional increase: gradual rise in W9 norm with moderate MSC levels.

The relationship between W9 norm (wavelet coefficient) and friction torque (τ_f_) was modeled using a second order polynomial fit:

(5) $$\tau_{f}=aW_{9}^{2}+bW_{9}+c$$

This correlation enabled direct estimation of friction severity from the *W9* coefficient, facilitating predictive maintenance.

#### Wavelet Selection and Frequency Analysis

In the Discrete Wavelet Transform framework, each decomposition level represents a specific portion of the signal frequency spectrum, enabling the separation of high-frequency electrical disturbances from lower-frequency electromechanical effects.

As summarized in Table 3, the first few levels of decomposition (W1–W4) primarily contain high-frequency components originating from switching operations, electrical noise, and measurement disturbances in the motor drive. The intermediate levels (W5–W7) capture mixed-frequency components that reflect a combination of electrical transients and motor commutation dynamics. In contrast, the higher decomposition levels (W8 and W9) correspond to low-frequency components associated with the electromechanical interaction between the motor and the load. These low-frequency components are particularly sensitive to gradual mechanical changes such as friction growth, load torque variations, and transmission resistance. Since such degradations influence the motor current indirectly through torque modulation, their effects become more pronounced in the higher-level wavelet coefficients. Consequently, the energy distribution in W8 and W9 provides meaningful information regarding the mechanical condition of the drive system.

Based on this frequency-domain interpretation, the higher-level coefficients were considered the most relevant indicators for condition monitoring, as they capture the electromechanical behavior of the system rather than high-frequency electrical disturbances.

### 2.3. Health Monitoring (Pre-Diagnostics) Algorithm Structure

The health monitoring algorithm, developed in MATLAB, classifies system health by evaluating extracted parameters (MSC peak, W8, and W9) against defined thresholds. The logical flow is as follows: 1. Signal acquisition → 2. Wavelet decomposition → 3. Feature extraction → 4. Threshold comparison → 5. Fault classification and alert generation.

**Figure 5:**
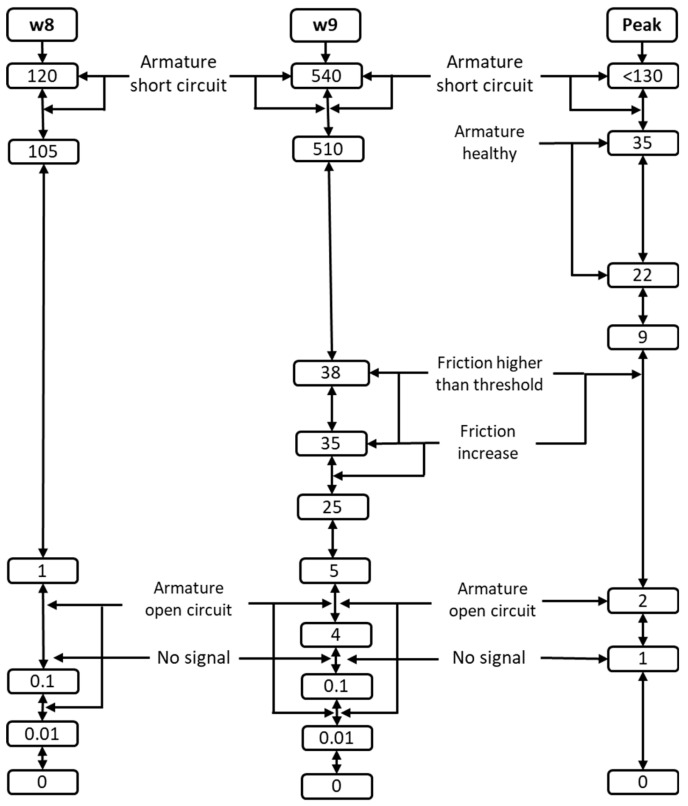
Threshold ranges of the extracted diagnostic features (MSC peak, W8, and W9) used for fault classification in the health monitoring algorithm.

In the fault classification stage, the extracted features were mapped into predefined diagnostic ranges to support consistent and robust decision-making. The threshold ranges of the wavelet-based features (W8 and W9) and the motor starting current peak are illustrated in Figure 5, which provides a clear visualization of feature separability under different operating and fault conditions. The figure highlights distinct value bands corresponding to healthy operation, electrical faults, and mechanical degradation. Electrical abnormalities are characterized by simultaneous increases in MSC peak and higher-level wavelet coefficients, whereas mechanical faults primarily manifest as progressive growth in the W9 coefficient with comparatively stable MSC amplitudes. By evaluating the combined position of a signal within the W8–W9–MSC feature space, the algorithm efficiently distinguishes overlapping fault signatures and improves classification reliability.

Fault categories include: armature healthy (ARH), armature short circuit (ASC), armature open circuit (AOC), friction increased maintenance required (FIMR), friction higher than threshold (FTH), and no signal/unidentified fault (UIF). The classification logic and feature thresholds are summarized in Table 4. Electrical faults are characterized by high MSC and W8–W9 values, while mechanical faults show moderate W9 growth with stable MSC amplitudes. The algorithm achieved rapid detection (<0.3 s) across all test cases, ensuring reliable early fault prediction.

Thresholds for each feature were defined from baseline simulations under healthy, degraded, and failed conditions. Electrical faults were primarily diagnosed by variations in MSC peak and W8/W9 amplitude, while progressive mechanical degradation was identified through increases in W9.

To ensure computational efficiency, two internal control variables were implemented:

- Count: tracks door actuation cycles for trend monitoring.
- Flag: controls diagnostic execution (Flag = 1 activates, Flag = 0 suspends).

**Figure 6:**
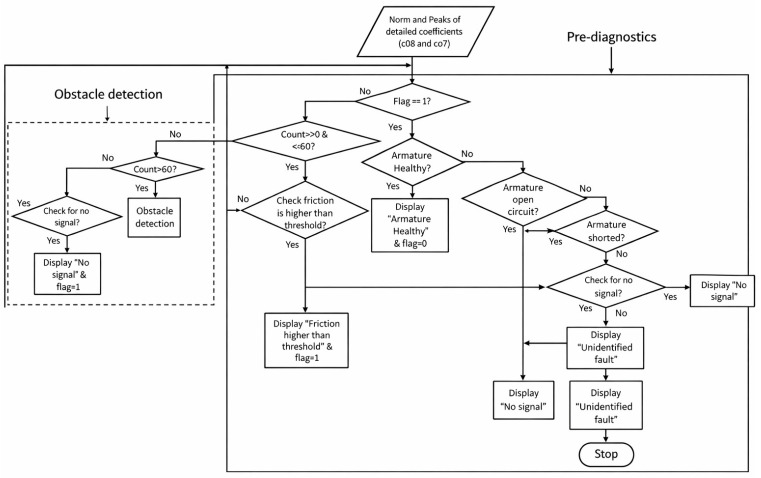
Flow chart of health monitoring (pre-diagnostics) and obstacle detection.

The diagnostic process begins with current signal acquisition during door closure, followed by signal denoising and nine-level DWT decomposition. The extracted features are then compared with their threshold values to determine the system state. When W9 exceeds 0.28 Nm equivalent, the algorithm triggers a friction above threshold alert, and halts door operation to prevent jamming or hardware damage. The complete logic flow is illustrated in Figure 6.

### 2.4. Threshold Calibration

The diagnostic thresholds for the extracted features wavelet coefficient norms (W8 and W9) and the motor starting current peak were determined using a combination of baseline simulations and experimental measurements. Initially, motor current signals were recorded under healthy operating conditions to establish reference feature distributions. The Discrete Wavelet Transform was applied to these signals, and the statistical characteristics of the extracted features were evaluated.

For each feature, the mean (μ) and standard deviation (σ) were calculated from the healthy baseline dataset. The decision thresholds were then defined using a statistical confidence margin to separate normal operational variations from abnormal fault conditions:

Threshold = μ + 3σ(6)

where μ represents the mean value of the feature under healthy conditions and σ denotes the corresponding standard deviation. The 3σ criterion was selected to provide a sufficient safety margin against measurement noise, signal variability, and minor operational disturbances.

After the statistical limits were first obtained from the simulation results, the threshold values were further checked using experimental data collected from the LabVIEW (version 19.0.1)-based test platform under healthy, degraded, and fault conditions. This two-step calibration process helped confirm that the selected thresholds could clearly distinguish between normal operation, electrical faults, and mechanical degradation without relying on a single dataset. In this proposed research, fixed threshold values were used to maintain algorithm stability and enable reliable real-time implementation.

### 2.5. Fault Classification Logic

A rule-based decision matrix was developed to automate fault identification based on feature thresholds. Diagnostic codes were assigned to enable integration with onboard monitoring systems indicated in Table 5. Correlation between MSC amplitude and W9 norm further allowed distinction between obstacle-induced spikes (MSC > 9 A) and genuine frictional growth (MSC < 9 A), minimizing false positives.

## 3. Experimental Setup and Validation

### 3.1. Experimental Configuration

**Figure 7:**
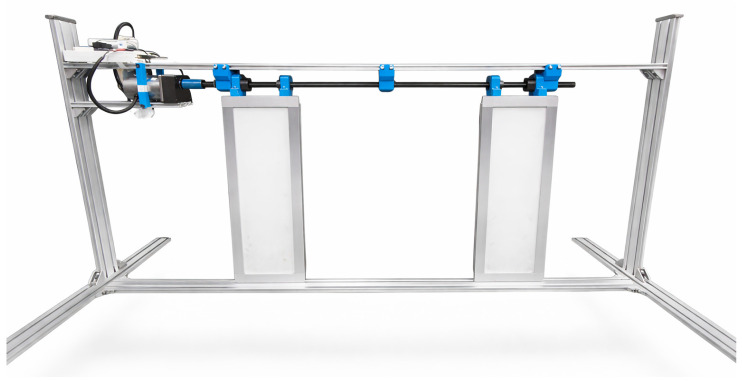
Scaled experimental prototype used for validation of the proposed health monitoring framework.

To validate the simulation outcomes, an experimental configuration was developed to validate the proposed health monitoring framework. Figure 7 shows the corresponding scaled experimental prototype constructed based on the CAD design. The setup consisted of a brushed DC motor, lead screw–roller assembly, and sliding door panels, replicating the kinematic and frictional behavior of a full-scale train door while permitting controlled fault emulation.

Motor current was monitored using a Hall effect current sensor with a 1 kHz sampling rate, and door displacement was recorded via a position feedback sensor for verification. Data acquisition and control were implemented through a DAQ interface integrated with the LabVIEW environment. The MATLAB-based pre-diagnostic algorithm was embedded within LabVIEW, facilitating real-time data processing, wavelet-based feature extraction, fault identification, and visual alert generation.

Three operational scenarios were examined to assess diagnostic accuracy:

1. Healthy Condition (Baseline): Nominal operation with minimal friction and no electrical faults.
2. Degraded Condition: Moderate frictional resistance and minor motor wear to represent early-stage degradation.
3. Fault Condition: Severe mechanical misalignment or electrical faults such as armature short and open circuits.

For each scenario, Discrete Wavelet Transform coefficients (W8, W9) and the motor starting current peak amplitude were extracted. Progressive increases in W9 and MSC amplitude were observed during early degradation, preceding obstacle-level forces. The system exhibited an average fault detection latency of <0.3 s, confirming the rapid response and sensitivity of the proposed pre-diagnostic framework.

### 3.2. Failure Mode and Effects Analysis

**Figure 8:**
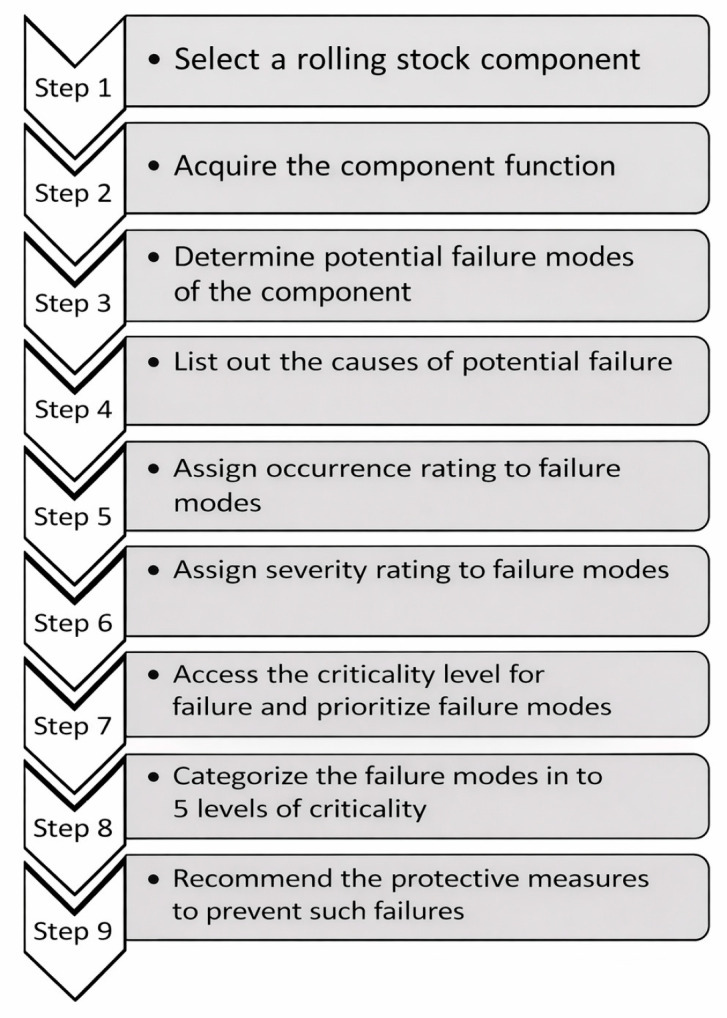
FMEA steps for rolling stock.

A comprehensive FMEA was conducted to evaluate potential failure risks in the train door actuation system. The assessment focused on four primary components: armature coil, gearbox, lead screw with bearings, and rollers. Each failure mode was analyzed using standard FMEA parameters: Occurrence (O), Severity (S), and Detection (D), with the Risk Priority Number (RPN = O × S × D) guiding the prioritization of maintenance actions. The analysis followed a nine-step procedure adapted from rolling-stock reliability studies of Dinmohammadi [24], as illustrated in Figure 8. The methodology included identification of failure mechanisms, effects on door operation, and assignment of preventive actions such as overcurrent protection, lubrication, or component replacement.

## 4. Results and Discussion

This section presents the results of both simulation and experimental validation for the proposed wavelet-based health monitoring (pre-diagnostic) framework. The analysis evaluates the system’s performance in accurately identifying mechanical and electrical faults in the train door actuation mechanism. Each fault scenario was examined in terms of detection accuracy, feature sensitivity, and diagnostic reliability, confirming the robustness of the developed methodology.

### 4.1. Simulation-Based Fault Analysis

A comprehensive simulation study was conducted with a torque command of 0.15 Nm to assess the system’s capability under varying operating and fault conditions. The algorithm continuously monitored wavelet coefficients (W8, W9) and motor starting current peak values to evaluate health status.

#### 4.1.1. Healthy Armature Condition

**Figure 9:**
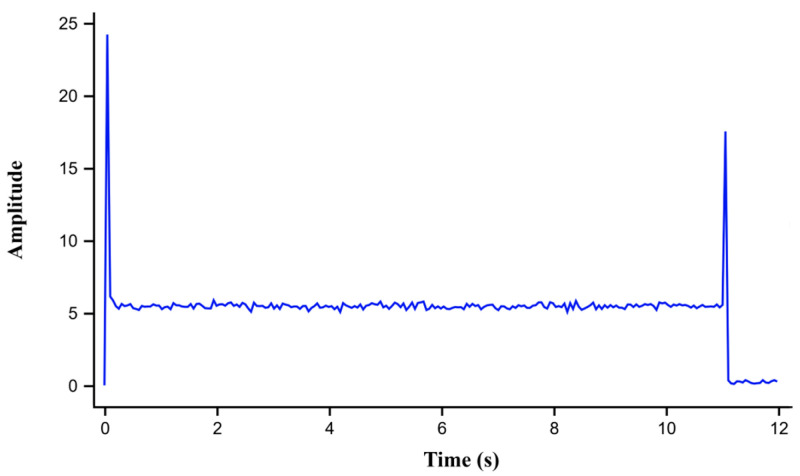
Simulation results of motor current profile and motor starting current (MSC) peak under healthy armature operation.

Under nominal operation, the current waveform exhibited distinct MSC peaks at the start and stop of the door motion (0 s and 11.1 s). The measured peak amplitude of 24.2 A was within the healthy threshold range (22 ≤ peak ≤ 35 A), confirming normal performance without triggering any warning. This established the baseline for healthy armature operation as shown in Figure 9.

#### 4.1.2. Frictional Faults

**Figure 10:**
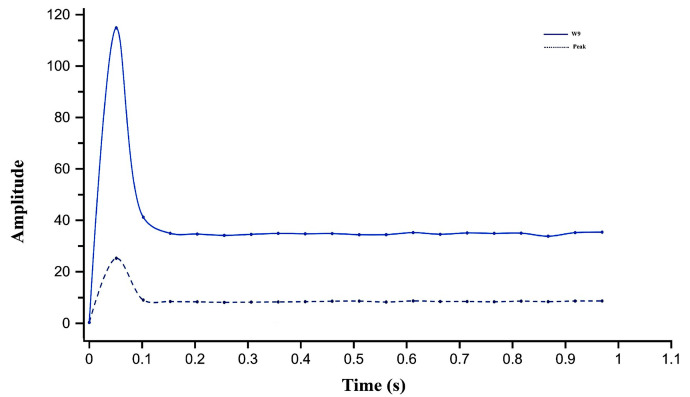
Simulation results of wavelet coefficient W9 and motor starting current peak under friction higher than threshold (FTH) condition.

The friction higher than threshold (FTH) fault was simulated next. Initially, the armature was detected as healthy in the first iteration. As the count exceeded 30, the function calculated friction using W9 and peak values to determine if it exceeded the threshold. At steady state, W9 and peak amplitudes reached 35.1 and 8.39, respectively, which fall within the FTH range shown in Figure 10.

**Figure 11:**
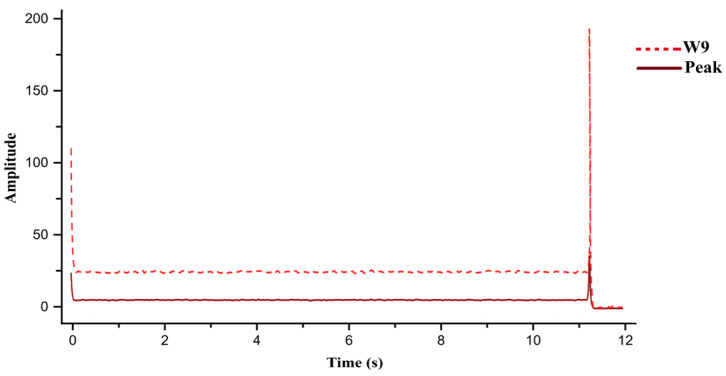
Simulation results of wavelet coefficient W9 and motor starting current peak under friction increased maintenance required (FIMR) condition.

Subsequently, the door friction increased (FIMR) fault was simulated. Again, the armature was initially healthy. When the count exceeded 60, friction was calculated from W9 and peak to check for an increase beyond the healthy threshold. Steady-state amplitudes of W9 and peak were 26 and 6, respectively, indicating the FIMR range as shown in Figure 11.

#### 4.1.3. Electrical Faults

##### Armature Short Circuit

**Figure 12:**
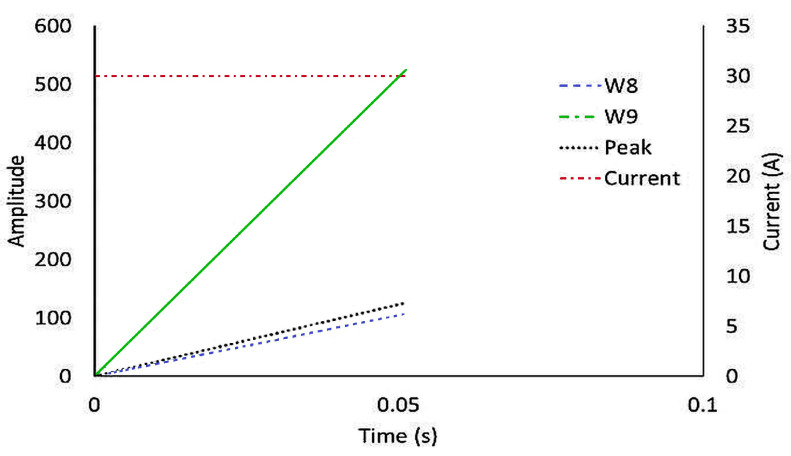
Simulation results of motor current waveform and wavelet-based features (W8 and W9) under armature short-circuit fault.

Introducing 0.01 Ω resistance caused an instantaneous current surge to 30 A. The resulting wavelet coefficients were W8 = 107.7, W9 = 525.6, and peak = 125.6, all exceeding the critical limits. The algorithm correctly identified the “armature short circuit” and terminated operation in Figure 12.

##### Armature Open Circuit:

**Figure 13:**
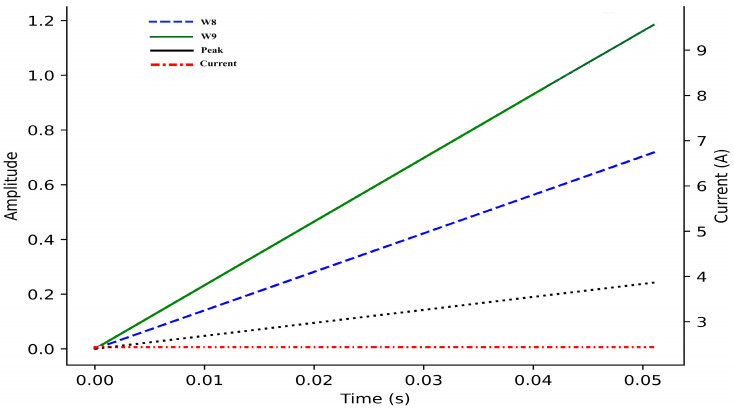
Simulation results of motor current waveform and wavelet-based features (W8 and W9) under armature open-circuit fault.

When the resistance was increased to 1 GΩ, current nearly dropped to zero, and corresponding values were W8 = 0.72, W9 = 1.19, and peak = 0.24, successfully classified as an armature open circuit in Figure 13.

These simulations confirmed that the algorithm accurately distinguished between mechanical degradation and electrical faults, achieving fast and consistent responses without false triggers.

### 4.2. Experimental Validation of Pre-Diagnostics

To verify the simulation results and assess the real-time performance of the proposed health monitoring (pre-diagnostic) algorithm, experimental validation was carried out using the LabVIEW-integrated test bench described in Section 3. The MATLAB-based diagnostic function was embedded within the LabVIEW environment through a data acquisition interface, enabling continuous signal monitoring and automated warning generation. The experimental procedure replicated all major fault scenarios: healthy operation, frictional degradation, and armature electrical faults.

#### 4.2.1. Healthy Armature Condition

**Figure 14:**
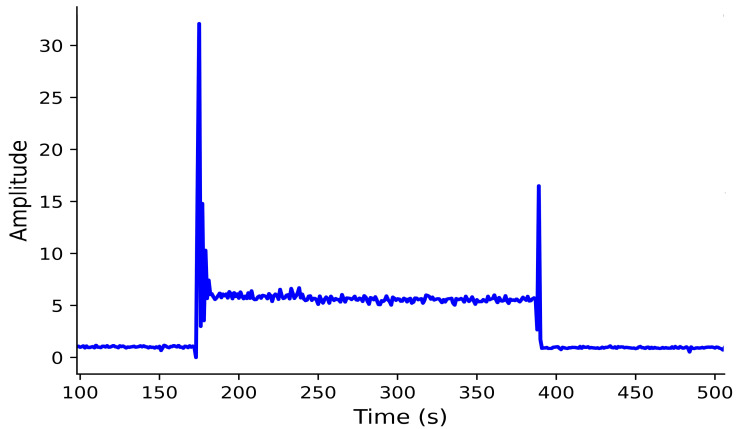
Experimental plot of MSC peak under healthy armature condition.

The baseline healthy condition was first evaluated by initiating the door closing operation using the control interface. As shown in Figure 14, the motor current exhibited a smooth transient profile, and the maximum signal coefficient peak amplitude reached 32.1 A at sample 175, slightly higher than the simulated value of 24.2 A. This deviation is attributed to inherent parameter tolerances in motor inductance, back electromotive force, and load inertia, which were estimated from datasheet data and measured resistance and speed. These parameter offsets were compensated within the MATLAB function by adjusting the detection thresholds. Consequently, the system correctly identified this condition as armature healthy, activating the corresponding LED indicator without triggering false alarms.

#### 4.2.2. Friction-Induced Faults

Two progressive friction fault conditions were emulated by manually applying opposing forces on the door panel during closure.

##### Friction Higher than Threshold (FTH)

**Figure 15:**
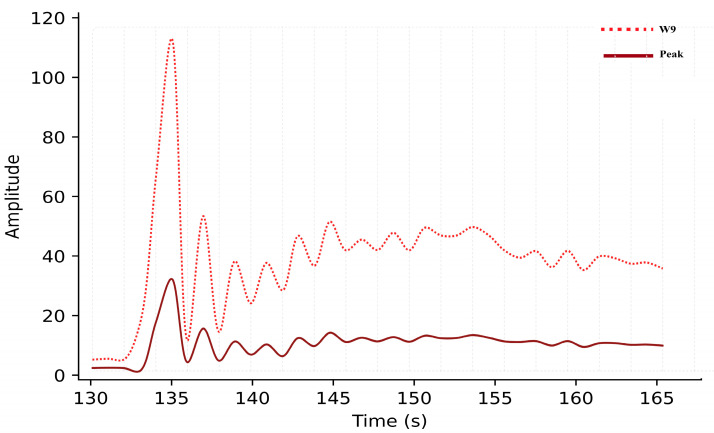
Experimental response under friction higher than threshold (FTH) condition of W9 and MSC peak.

When a moderate opposing force was applied, the diagnostic algorithm detected a notable rise in the amplitude of wavelet coefficient W9 = 37.4 and MSC peak = 8.83, as shown in Figure 15. These values exceeded the upper friction threshold predicted by the polynomial fitting model derived. The system immediately issued a “friction higher than threshold” alert, activated the FTH indicator, and automatically halted door movement to prevent potential jamming or actuator overload.

##### Friction Increased Maintenance Required (FIMR)

**Figure 16:**
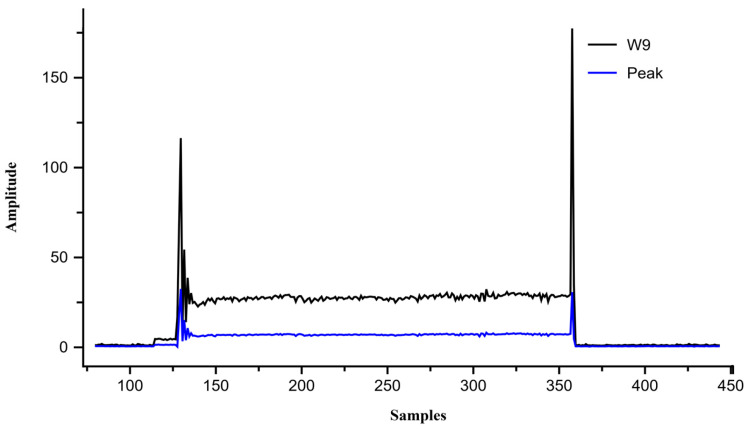
Experimental response of W9 and MSC peak under friction increased maintenance required (FIMR) condition.

A light opposing force was then applied to simulate early-stage degradation. The algorithm detected an incremental rise to W9 = 26.7 and Peak = 6.49 as shown in Figure 16. These values fell within the defined maintenance range (0.21–0.28 Nm). The system displayed a “friction increased maintenance required” warning, signaling the need for inspection while allowing continued operation. These experiments confirm the sensitivity of W9 and peak amplitude trends to gradual frictional changes, validating the algorithm’s predictive capability in identifying incipient mechanical degradation before failure.

#### 4.2.3. Armature Electrical Faults

To further evaluate electrical fault detection accuracy, both armature short-circuit and open-circuit faults were experimentally emulated.

##### Short-Circuit Fault

**Figure 17:**
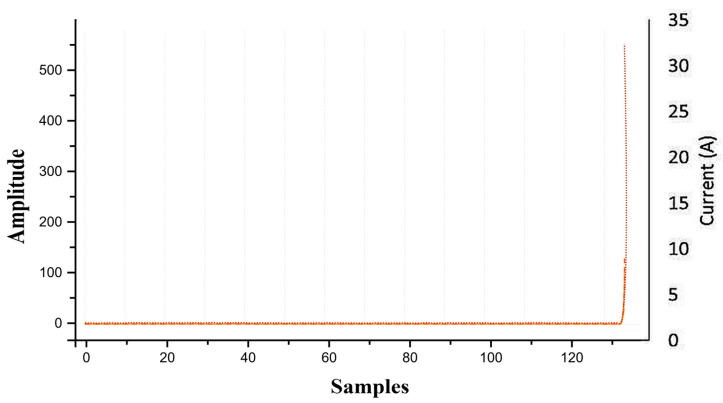
Experimental validation of armature short-circuit fault using wavelet energy features and MSC peak.

Since laboratory conditions limited the maximum supply current, short-circuit conditions were simulated by feeding pre-recorded 30 A current samples into the DAQ system. Upon detecting these samples at iteration 135, the algorithm calculated W8 = 109, W9 = 564, and peak = 138 as shown in Figure 17. These high-magnitude signatures exceeded all threshold limits, resulting in an “armature short circuit” alert. The system immediately terminated door motion and displayed the fault condition on the user interface.

##### Open-Circuit Fault

**Figure 18:**
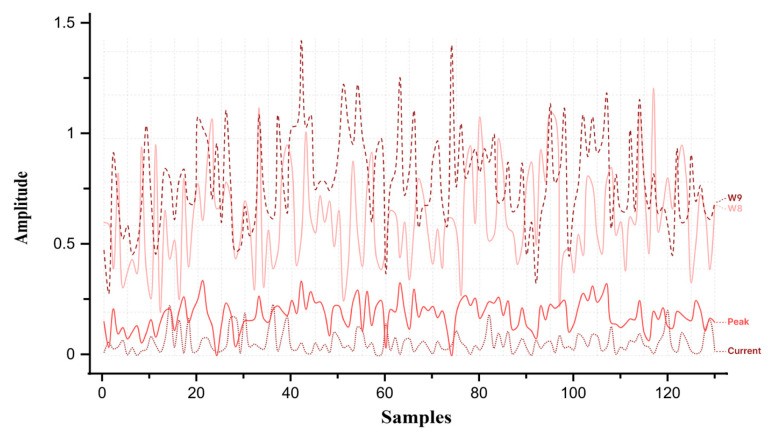
Experimental validation of armature open-circuit fault using wavelet energy features and MSC peak.

For open-circuit testing, one terminal of the motor was disconnected from the driver while executing the closing command. The measured current was near zero, with minimal wavelet amplitudes (W8 = 0.7, W9 = 1.2, peak = 0.24) due to residual sensor noise as shown in Figure 18. These values aligned with open-circuit thresholds, prompting the “armature open circuit” warning and immediate operation halt. The accurate distinction between short- and open-circuit faults in real time validates the robustness and reliability of the proposed wavelet-based health monitoring (pre-diagnostic) system for both electrical and mechanical failures.

#### 4.2.4. Statistical Validation of Experimental Results

To evaluate the consistency of the experimental results, each operating condition was tested three times following standard engineering practice. During each trial, the key features, namely the motor starting current peak and the wavelet coefficients W8 and W9, were recorded. The results are summarized in Table 6, using mean and standard deviation to show how much variation exists between the trials.

The results indicate that the variation across trials is minimal for all operating conditions, as reflected by the low standard deviation values. This demonstrates that the extracted features are consistent and repeatable under both healthy and faulty states. Moreover, a clear distinction can be observed between different fault conditions based on the feature values, confirming that the selected parameters effectively differentiate between electrical and mechanical faults. Overall, the small variation within each condition and the clear separation between different conditions support the reliability of the proposed method.

These findings also highlight the practical strength of the proposed approach. While advanced methods such as deep learning and Bayesian inference offer powerful diagnostic capabilities, they often involve higher computational complexity and limited interpretability. In contrast, the proposed method achieves a balance between accuracy, interpretability, and practical implementation. The use of wavelet-based features provides clear physical insight into fault behavior, and the low computational requirement enables real-time application. Furthermore, the consistent experimental performance demonstrates the robustness of the approach under practical operating conditions.

### 4.3. Fault Classification Performance

The classification performance of the proposed diagnostic algorithm was evaluated across different fault conditions. Table 7 indicates results of high detection accuracy for all operating states, including 98.2% for armature healthy conditions, 100% for armature short-circuit faults, 99.1% for armature open-circuit faults, 96.7% for friction increase, and 97.8% for friction-above-threshold conditions. These results demonstrate the reliability of the proposed wavelet-based monitoring framework in accurately distinguishing between electrical and mechanical faults. A confusion matrix is presented to illustrate the classification performance of the algorithm.

### 4.4. Failure Mode and Effects Analysis Results and Discussion

The FMEA revealed that armature coil faults, particularly short- and open-circuit failures, exhibited the highest criticality and require immediate preventive measures including overcurrent protection and thermal monitoring. Mechanical faults showed moderate values such as gearbox tooth damage, lead screw misalignment, and roller seizure indicating the need for periodic lubrication, alignment checks, and replacement where necessary. Potential failure modes were identified, including armature short/open circuits, gearbox tooth breakage, lead screw misalignment, and roller seizure, with causes such as insulation breakdown, wear, friction, dust, corrosion, and overload. Using historical data from 38 Class 380 EMU trains, Occurrence (O) and Severity (S) ratings were assigned, and the risk factor (R = O × S) was calculated to prioritize failures. Failures were categorized into five criticality levels, and preventive measures of overcurrent protection, component replacement, and regular lubrication were recommended. The FMEA successfully quantified the risk associated with door actuation faults (Table 8).

The Failure Mode and Effects Analysis was used not only to evaluate potential risks in the train door actuation system but also to guide the development of the proposed diagnostic framework. Based on the FMEA results, faults with higher values, such as armature electrical faults and lead screw misalignment, were prioritized during simulation and experimental validation. These failure modes directly influence the motor current characteristics and therefore served as the primary targets for feature extraction and threshold calibration. For example, electrical faults affecting the armature coil were associated with significant changes in motor starting current, while mechanical faults related to friction growth in the lead screw and rollers produced observable variations in the low-frequency wavelet coefficients (W8 and W9). Consequently, the FMEA analysis guided the selection of diagnostic features and supported the establishment of threshold values used in the proposed pre-diagnostic monitoring framework.

### 4.5. Limitations and Generalizability of Experimental Validation

Although the experimental results show that the proposed method works well, there are some limitations in how the faults were created in the lab. Specifically, the armature short-circuit fault was not physically produced; as an alternative, pre-recorded current signals were used. Similarly, the open-circuit condition was implemented as a complete disconnection of the motor terminal. While these methods are safe and easy to control, they do not fully represent how faults actually develop in real systems, where they often appear gradually or intermittently. Because of this, the fault signals obtained in the experiments may be more ideal and clearer than those found in real operating conditions. In actual train door systems, faults usually develop slowly and are affected by load changes, environmental conditions, and noise. Therefore, even though the proposed method shows good performance in detecting faults, its performance in real-world conditions may vary.

However, the use of wavelet features and motor current signals still provides a strong basis for detecting abnormal behavior. With proper tuning and adjustment of thresholds, the method can be applied to real systems. In future work, more realistic fault conditions and real-time data from operating systems will be considered to further validate and improve the approach.

### 4.6. Comparison of the Proposed Method with Conventional Monitoring Techniques

To further evaluate the practical significance of the proposed monitoring framework, it is useful to compare its characteristics with commonly used monitoring approaches reported in railway door systems and motor driven mechanisms. These approaches include sensor-based obstacle detection, vision-based monitoring using deep learning, conventional current threshold monitoring, and frequency-domain signal analysis. Table 9 summarizes the key differences between these approaches and the proposed method in terms of monitoring signals, capabilities, and limitations.

Conventional sensor-based and vision-based approaches require additional hardware components such as infrared sensors or camera systems, which increase system complexity and maintenance requirements. Traditional current threshold monitoring methods are able to detect severe faults but often lack sensitivity to gradual degradation. In contrast, the proposed method utilizes wavelet-based analysis of motor current signals to capture transient electromechanical behaviors associated with friction growth and electrical abnormalities. This enables earlier detection of subtle degradation indicators, supporting predictive maintenance without requiring additional sensing infrastructure.

## 5. Conclusions

This study demonstrates that the proposed wavelet-transform-based monitoring framework enables pre-diagnostic detection, meaning that early degradation signatures can be identified before functional failure or severe mechanical resistance occurs. By analyzing motor current signals, the system extracts key features, namely the W8 and W9 coefficients and motor starting current, to differentiate between healthy operation, frictional degradation, and armature faults. Simulation results demonstrated accurate identification of both friction-related and electrical abnormalities, with predictive alerts generated before operational failure. The method successfully identified armature short and open circuits, while friction growth beyond threshold limits triggered maintenance warnings, ensuring safe and reliable door operation.

Experimental validation using a LabVIEW-integrated test bench confirmed the simulation results, with detection latency below 0.3 s and strong correlation between measured signals and simulated thresholds. The algorithm effectively distinguished between obstacle-induced and frictional disturbances, achieving high diagnostic accuracy under real-world conditions. By integrating Failure Mode and Effects Analysis, critical components such as the armature, gearbox, and lead screw were prioritized for preventive maintenance. Compared with conventional threshold-based or sensor-dependent monitoring methods, the proposed approach demonstrates higher sensitivity to subtle current variations associated with early friction growth and armature abnormalities. This improved sensitivity enables earlier detection of degradation, increasing the prediction lead time for maintenance decisions and allowing corrective actions before operational faults occur. Consequently, the method provides a cost-effective, sensorless solution that supports predictive maintenance and improves the reliability and safety of train door systems.

## Figures and Tables

**Table 1:** Summary of related works and identified research gaps.

Study	Method	Application	Key Contribution	Research Gap
Tan et al. [2]	Deep learning (DDSNet)	Train door obstacle detection	High-accuracy object detection in complex environments	Requires cameras, high computational cost, sensitive to lighting and occlusion
Liu et al. [3]	AnoDet (vision-based)	Railway platform monitoring	High-precision anomaly detection (>97%)	Depends on visual data, not suitable for embedded real-time systems
Yan et al. [4]	Deep learning detection	Metro door systems	Accurate large-object detection	High hardware and processing requirements
Liu et al. [6]	Back-EMF analysis	Motor fault detection	Detection of stall conditions	Limited sensitivity to early-stage faults
Michal et al. [7], Li et al. [8]	Current ripple analysis	DC motor monitoring	Detection of obstruction events	Affected by noise, limited robustness
Miljkovic [15]	MCSA review	Electrical machine diagnostics	Non-intrusive fault detection	Mostly focused on steady-state faults
Wu et al. [14]	Wavelet-based analysis	Railway systems	Improved fault detection using wavelets	Focus on post-fault detection, not early prediction
Abbaszadeh et al. [20]	Wavelet transform	Induction motors	Broken rotor bar detection	Not applied to complex electromechanical systems
Teel-Jongebloed [24]	Statistical current analysis	DC motor degradation	Detection of brush wear	Limited integration of mechanical faults

**Table 2:** L1-norm at different decomposition levels.

L1-Norm	Friction Increase (0.28 Nm)	Armature Short Circuit (0.01 Ω)	Armature Open Circuit (1 GΩ)
W1	0.0108	4.7	6.2
W2	0.0168	8.3	4.9
W3	0.0256	13.4	6.8
W4	0.0299	15.6	7.6
W5	0.0554	17.9	4.2
W6	0.1357	5.1	3.2
W7	0.6551	12.7	1.9
W8	6.7123	112.6	0.7
W9	32.6066	543.4	1.2

**Table 3:** Frequency band interpretation of wavelet decomposition levels for motor current signal analysis.

Level	Frequency Band
W1–W4	Switching noise
W5–W7	Mixed electrical effects
W8–W9	Mechanical load and friction variations

**Table 4:** Feature–threshold combinations used for fault classification in the proposed algorithm.

Feature	Threshold	W8	W9	Peak	Door Operation Status
Armature healthy	1 Ω			22 ≥ Peak ≤ 35	Door can be operated
Armature open circuit	1 GΩ	0.01 > W8 < 1	0.01 > W9 < 5	≤2	Door out of order
Armature short circuit	0.01 Ω	105 > W8 ≤ 120	510 > W9 ≤ 540	≤130	Door out of order
Friction increased warning	0.21–0.28		25 > W9 ≤ 35	<9	Door can be operated
Friction higher than threshold level	≥0.29		35 ≥ W9 ≤ 38	<9	Door out of order
No signal		0.1 > W8 < 1	0.1 > W9 < 4	≤1	Door not in operation
Unidentified fault					Door out of order

**Table 5:** Feature detection codes.

Feature	y	p	q
ARH		50	
AOC	100	20	
ASC	100	30	
NO SIGNAL	40		
UIF	100	40	
FTH	100		40
FIMR			10

**Table 6:** Statistical analysis of extracted features under different conditions.

Condition	Feature	Trial 1	Trial 2	Trial 3	Mean ± Standard Dev.
**Healthy Armature**	MSC Peak	32.12	31.94	32.31	32.12 ± 0.19
**Friction Higher Than Threshold**	MSC Peak	6.49	6.35	6.58	6.47 ± 0.12
	W9	26.75	27.0	26.32	26.69 ± 0.34
**Friction Increased Maintenance Required**	MSC Peak	8.83	8.75	8.90	8.83 ± 0.08
	W9	37.44	37.82	37.16	37.47 ± 0.33
**Armature Short Circuit**	MSC Peak	138	137	138	137.67 ± 0.58
	W8	109	110	108	109.0 ± 1.00
	W9	563	567	564	564.67 ± 2.08
**Armature Open Circuit**	MSC Peak	0.24	0.22	0.25	0.24 ± 0.02
	W8	0.71	0.69	0.70	0.70 ± 0.01
	W9	1.22	1.15	1.24	1.20 ± 0.05

**Table 7:** Detection accuracy of the proposed diagnostic algorithm under different fault conditions.

Fault Type	Detection Rate
Armature Healthy	98.2%
Armature Short Circuit	100%
Armature Open Circuit	99.1%
Friction Increase	96.7%
Friction Above Threshold	97.8%

**Table 8:** FMEA summary for train door actuation system.

Component	Function	Potential Failure Mode	Effect	S	O	R	Preventive Action
Armature Coil	Converts electrical to mechanical torque	Short/open circuit	No motion, reduced speed	7	4	28	Overcurrent protection
Gearbox	Transmits and amplifies torque	Broken tooth	Reduced torque, vibration	8	2	16	Replacement
Lead Screw & Bearings	Convert rotary to linear motion	Misalignment	Increased friction, wear	8	3	24	Lubrication, alignment
Rollers	Provide sliding motion	Roller seizure	Jerky motion, noise	4	3	12	Lubrication, replacement

**Table 9:** Comparison between the proposed monitoring approach and commonly used fault detection methods.

Method	Monitoring Signal	Main Capability	Limitations	Advantage of Proposed Method
Sensor-based obstacle detection (IR/edge sensors)	External sensors	Detects physical obstruction during door closure	Requires additional hardware, frequent calibration, sensitive to environmental conditions	Proposed method uses existing motor current signals without additional sensors
Vision-based deep learning detection	Camera images	High accuracy in detecting foreign objects near doors	High computational cost, lighting dependency, occlusion problems	Proposed approach avoids cameras and enables real-time monitoring with lower computational requirements
Current threshold monitoring	Motor current amplitude	Detects large electrical faults and severe obstructions	Low sensitivity to gradual mechanical degradation	Wavelet features improve sensitivity to small current variations
Frequency-domain current analysis	Motor current spectrum	Identifies electrical anomalies in motors	Limited capability for transient and non-stationary signals	Wavelet transform captures transient electromechanical behaviors
Wavelet-based fault detection (existing studies)	Wavelet coefficients	Detects faults after system performance degradation	Primarily focused on post-fault detection rather than early monitoring	Proposed method enables pre-diagnostic detection of early degradation
Proposed method	Motor current + wavelet coefficients (W8, W9) + MSC	Early detection of mechanical friction growth and armature faults		Improved sensitivity to subtle degradation and increased prediction lead time without additional sensors

## Data Availability

Data are contained within the article.
